# Enhancing procedural quality and efficiency of methacholine bronchial challenge tests in patients with suspected cough-variant asthma: a quasi-experimental study utilizing nurse–patient communication and respiratory training

**DOI:** 10.3389/fmed.2026.1879079

**Published:** 2026-07-01

**Authors:** Yu Hu, Hailun Wang, Chang Tan, Yu He, Jin Zhu, Shi Chen, Tian Zhao, Enhong Liu, Ying Chen, Sisi Chen, Beibei Zhang, Hongmei Xiang, Fajiu Li, Xuecun Wang

**Affiliations:** 1 Department of Respiratory and Critical Care Medicine, The Sixth Hospital of Wuhan, Affiliated Hospital of Jianghan University, Wuhan, Hubei, China; 2 The Sixth Hospital of Wuhan, Affiliated Hospital of Jianghan University, Wuhan, Hubei, China

**Keywords:** bronchial challenge test, COMFORT model, cough-variant asthma, maneuver quality, patient compliance, procedural quality, procedural reliability, spirometry

## Abstract

**Objective:**

Methacholine bronchial challenge testing (MBCT) is important for documenting airway hyperresponsiveness in patients with suspected cough-variant asthma (CVA), yet its procedural quality may be compromised by suboptimal patient maneuver execution and procedure-related anxiety. This study evaluated whether COMFORT-based nurse–patient communication combined with brief mechanical respiratory training improves MBCT procedural quality.

**Methods:**

This single-center quasi-experimental study with historical controls followed TREND guidance. Ninety-four adults with suspected CVA were sequentially enrolled: 50 controls received standard visual/verbal instruction, and 44 patients received COMFORT-based counseling plus supervised inspiratory/expiratory training before MBCT; one intervention patient dropped out. Propensity score matching (PSM) was used to reduce temporal confounding. Outcomes included first-attempt success rate, in-chamber testing duration, spirometric maneuver quality, SAS/SDS scores, MBCT positivity, and adverse events.

**Results:**

In the matched cohort (41 pairs), baseline characteristics were well-balanced after PSM. The intervention group showed a higher first-attempt success rate than the control group (75.6% vs. 24.4%, *p* < 0.001) and a shorter in-chamber testing duration (11.85 ± 3.12 min vs. 16.90 ± 3.85 min, p < 0.001). Optimal FVC and FEV1 values were also higher in the intervention group (FVC: 2.95 ± 0.32 L vs. 2.58 ± 0.35 L; FEV1: 2.05 ± 0.25 L vs. 1.70 ± 0.28 L; both *p* < 0.001), consistent with improved maneuver execution. Post-procedure SAS and SDS scores were lower in the intervention group (SAS: 35.10 ± 7.85 vs. 39.50 ± 8.12, *p* = 0.015; SDS: 30.05 ± 5.90 vs. 35.80 ± 6.25, *p* < 0.001). Mild procedure-related adverse events were less frequent in the intervention group (7.3% vs. 24.4%, *p* = 0.032). The MBCT positivity rate was numerically higher in the intervention group but did not differ significantly between groups (29.3% vs. 17.1%, *p* = 0.190).

**Conclusion:**

Integrating empathetic nurse–patient communication with targeted respiratory mechanical training significantly improves the procedural quality of MBCTs. This combined approach improved maneuver execution and first-attempt success and reduced in-chamber testing duration, while requiring additional pre-test preparation time. These findings support a procedural-quality intervention for MBCT rather than evidence of improved diagnostic accuracy or immediate physiological improvement in lung function.

## Graphical abstract

**Figure:**
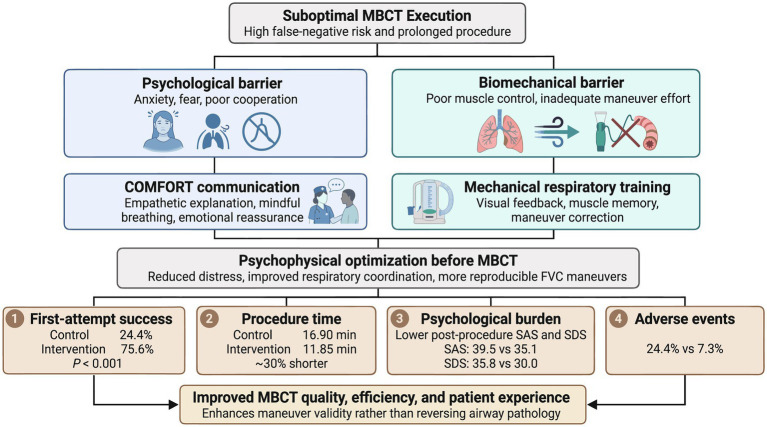
Mechanistic graphical abstract of COMFORT-based communication and respiratory training before MBCT. Psychological reassurance and respiratory maneuver rehearsal jointly reduce distress and correct suboptimal breathing mechanics before methacholine bronchial challenge testing. These effects improve first-attempt maneuver success, shorten testing time, and reduce mild procedure-related adverse events, while the observed spirometric gains reflect improved effort and maneuver quality rather than immediate physiological reversal of airway dysfunction.

## 1 Introduction

Accurate assessment of dynamic lung volumes and airway hyperresponsiveness (AHR) via pulmonary function testing is paramount for the diagnosis and longitudinal management of obstructive airway diseases (1, 2). According to the Global Initiative for Asthma (GINA) guidelines, confirming the diagnosis of asthma necessitates both a history of variable respiratory symptoms and objective demonstration of variable expiratory airflow limitation (2, 3). While classical asthma presents with wheezing and dyspnea, diagnosing atypical phenotypes, particularly cough-variant asthma (CVA), presents profound clinical challenges. CVA is characterized by chronic, non-productive cough as the sole or predominant symptom, often presenting with normal baseline spirometry (4, 5). Consequently, when baseline spirometry is normal or nondiagnostic, identifying AHR through a methacholine bronchial challenge test (MBCT) is a recommended approach for documenting airway hyperresponsiveness in patients with suspected CVA (6). Previous evidence has underscored the critical role of bronchial hyperresponsiveness testing, with methacholine challenge demonstrating high diagnostic sensitivity and specificity for CVA (7).

Despite its clinical utility, the execution of MBCT is technically demanding and heavily reliant on patient cooperation. The European Respiratory Society (ERS) technical standard for bronchial challenge testing strictly stipulates the necessity of forceful, maximal, and reproducible expiratory maneuvers (8). Unfortunately, suboptimal patient effort—often secondary to poor comprehension of the maneuver, insufficient respiratory muscle coordination, or psychogenic hesitation—frequently results in artifactual data (9). Such procedural failures may produce unacceptable or indeterminate spirometric curves and may underestimate airway responsiveness, thereby necessitating repeated attempts or repeat testing (10).

Patients undergoing MBCT, particularly those with prolonged undiagnosed chronic cough like CVA, often harbor severe baseline anxiety (11). This psychological burden is exacerbated by the fear of the testing apparatus and the apprehension of pharmacologically induced bronchoconstriction (12). Conventional pre-test counseling typically involves rudimentary verbal instructions or brief video demonstrations. This paradigm systematically neglects the bidirectional interplay between psychological distress and physical performance, often failing to adequately reassure the patient or correctly calibrate their respiratory mechanics (13). When patients are anxious, their respiratory pattern shifts towards shallow, rapid thoracic breathing, which intrinsically inhibits their ability to generate the deep inspiratory capacities and explosive expiratory flows required by ERS spirometry criteria (14).

To address these intertwined psychological and mechanical barriers, multidisciplinary interventions are urgently required. We hypothesize that these interventions act synergistically: psychological reassurance mitigates performance-inhibiting anxiety, which in turn facilitates better mechanical execution; conversely, successfully mastering the mechanical technique through practice builds patient confidence and further reduces testing anxiety. From a psychological perspective, structured nurse–patient communication frameworks, such as the COMFORT model (Communication, Orientation and opportunity, Mindfulness, Family, Ongoing, Reiterative, and Team), have demonstrated robust efficacy in alleviating clinical anxiety and fostering patient trust in acute care settings (15, 16). Concurrently, from a biomechanical perspective, targeted use of visual-feedback respiratory trainers prior to testing can serve as an effective “warm-up.” These devices engage diaphragmatic breathing, strengthen transient respiratory muscle memory, and translate abstract verbal instructions (“blow out hard and fast”) into tangible, visually verifiable goals (17, 18).

While previous studies have highlighted the isolated benefits of psychological support (19) or long-term respiratory rehabilitation in chronic asthma management (20), there is a critical lacuna in the literature regarding the acute application of combined psychophysical interventions immediately preceding diagnostic MBCT. Furthermore, previous studies have often conflated short-term improvements in test performance with true physiological disease resolution, leading to misinterpretations of data (21).

Therefore, the primary objective of this quasi-experimental study was to evaluate the clinical utility of implementing the COMFORT communication model paired with brief mechanical respiratory training prior to MBCT in patients with suspected CVA. We hypothesized that this dual psychophysical intervention would: (1) significantly improve procedural quality, reflected by higher first-attempt success rates and shorter in-chamber testing duration; (2) optimize spirometric maneuver execution (increased maximal effort generation); and (3) significantly attenuate procedure-induced psychological distress and related somatic adverse events.

## 2 Materials and methods

### 2.1 Study design and ethical considerations

This study utilized a single-center, quasi-experimental design with historical controls, strictly adhering to the Transparent Reporting of Evaluations with Nonrandomized Designs (TREND) statement (22). Due to the logistical constraints of implementing a new standard operating procedure (SOP) across the respiratory testing unit, a time-based sequential allocation approach was adopted. The study protocol was reviewed and approved by the Ethics Committee of The Sixth Hospital of Wuhan, Affiliated Hospital of Jianghan University. Written informed consent was obtained from all participants prior to enrollment. All procedures were conducted in accordance with the Declaration of Helsinki.

### 2.2 Participants and setting

Patients scheduled for methacholine bronchial challenge testing (MBCT) at the Department of Respiratory and Critical Care Medicine between March 2024 and July 2024 were evaluated for enrollment. Inclusion criteria were: (1) adults aged 18 to 75 years; (2) clinical suspicion of CVA, defined as chronic cough lasting >8 weeks without obvious etiology and systematic exclusion of alternative causes such as gastroesophageal reflux disease, upper airway cough syndrome, and angiotensin-converting enzyme inhibitor use; (3) baseline forced expiratory volume in 1 s (FEV1) to forced vital capacity (FVC) ratio (FEV1/FVC) ≥ 70% and FEV1 ≥ 80% of predicted value, ensuring the safety prerequisites for ERS methacholine challenge guidelines (8); and (4) adequate cognitive ability to understand instructions. Exclusion criteria included: (1) classic asthma symptoms (e.g., wheezing); (2) absolute contraindications to MBCT (e.g., recent myocardial infarction, uncontrolled hypertension, known aortic aneurysm); (3) recent respiratory tract infection within the past 4 weeks; and (4) inability to comprehend Mandarin Chinese.

Alternative causes were screened using a structured history and chart review, including medication exposure to angiotensin-converting enzyme inhibitors, symptoms suggestive of upper airway cough syndrome or rhinosinusitis, reflux-related symptoms, recent respiratory infection, and chest imaging when clinically indicated. Patients with an alternative primary explanation for chronic cough were not enrolled.

**Figure 1:**
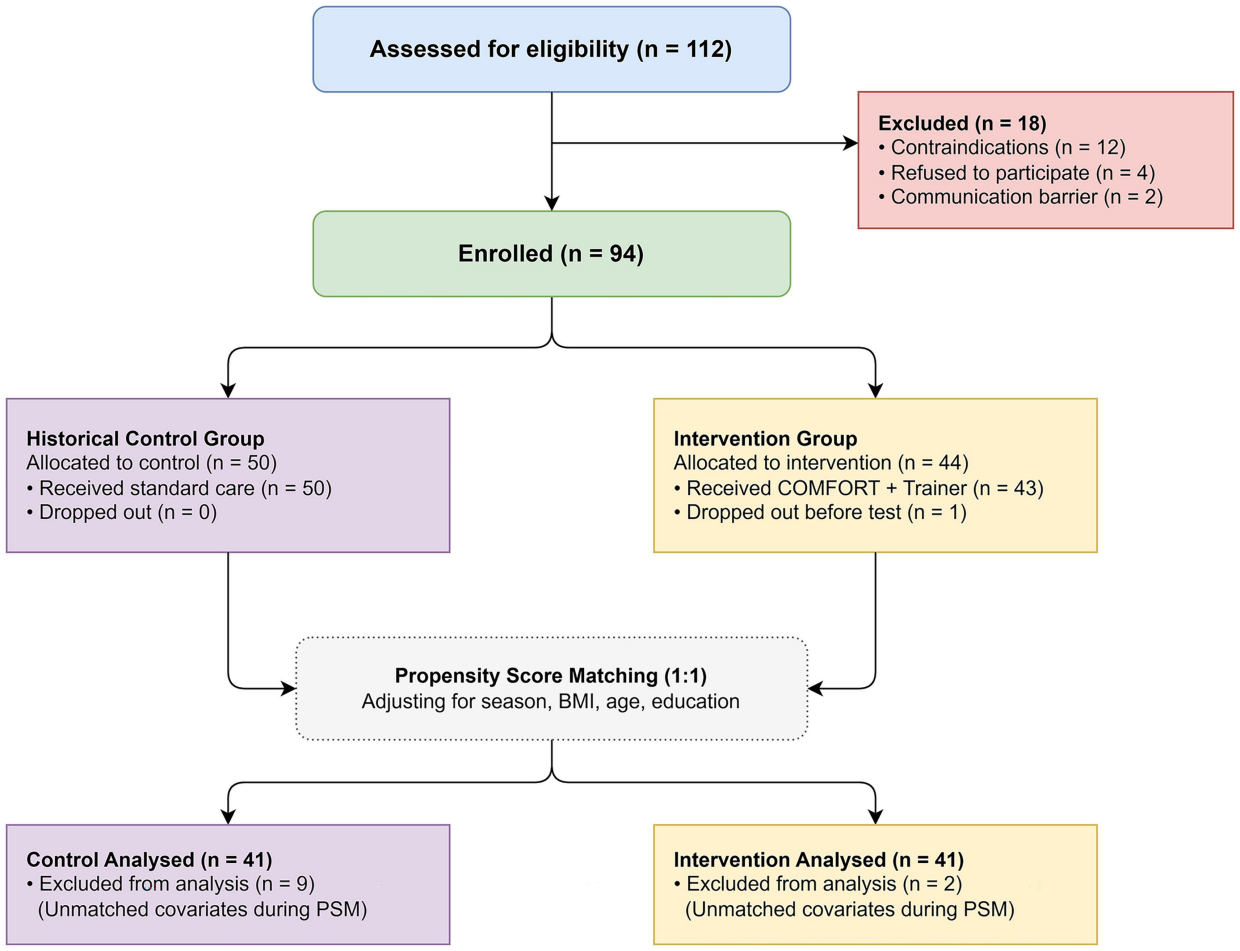
Modified CONSORT flow diagram of participant enrollment and allocation. Demonstrates the sequential allocation and Propensity Score Matching (PSM) process to account for seasonal variation.

To prevent contamination between study arms, a temporal sequence allocation was employed. Patients recruited between March 2 and April 18, 2024, formed the historical control group. Following a one-week departmental transition and staff training period, patients recruited between April 19 and July 15, 2024, formed the prospective intervention group (Figure 1).

### 2.3 Sample size calculation

*A priori* sample size calculation was performed using G*Power software (Version 3.1.9.7). Based on preliminary departmental data, we anticipated a medium-to-large effect size (Cohen’s *d* = 0.55) regarding the reduction of in-chamber testing duration. To achieve 80% statistical power (1-*β*) with a two-tailed alpha level of 0.05, a minimum of 106 subjects (53 per group) was required. Anticipating a 10% dropout rate, we aimed to enroll approximately 118 patients. Ultimately, 112 patients were screened, and 94 were deemed eligible and enrolled. The achieved sample size was below the *a priori* target; therefore, effect estimates, particularly for secondary outcomes, were interpreted cautiously and with attention to 95% confidence intervals.

### 2.4 Standard preparations and control protocol

Strict medication washout periods were enforced uniformly across both cohorts in accordance with ERS guidelines: short-acting β2-agonists (SABA) for 8 h, short-acting muscarinic antagonists (SAMA) for 24 h, long-acting bronchodilators (LABA/LAMA) for 48 h, and antihistamines for 72 h (8). Consumption of caffeine, vigorous exercise, and smoking were prohibited for 4 h pre-test.

The historical control group received the standard of care. This comprised a 5-min pre-test period where a technician provided standardized verbal instructions and demonstrated the maneuver using a pictorial guide. MBCT was performed utilizing the MasterScreen-PFT spirometer (Jaeger, Germany) via the dosimeter method. Aerosolized methacholine was inhaled incrementally (concentrations ranging from 0.0625 to 16 mg/mL). The test was considered positive if a ≥ 20% drop in FEV1 (PC20) was observed.

### 2.5 Comprehensive intervention protocol

Patients in the intervention cohort received a combined psychophysical intervention immediately prior to the standard MBCT. Importantly, the time spent on this intervention was distinct from and not included in the “total procedural duration” metric of the MBCT itself, allowing comparison of in-chamber test execution time; total patient preparation time and nursing resource use were considered separately in the Discussion.

#### 2.5.1 Psychological intervention: the COMFORT model

A specialized respiratory nursing team implemented the COMFORT communication framework (15) during a dedicated 15-min pre-test counseling session. (1) Communication & Orientation: Nurses utilized clear, jargon-free language to explain the physiology of the test, demystifying the “tight chest” sensation caused by methacholine as a safe and reversible diagnostic step. (2) Mindfulness: Patients were guided through 2 min of mindful, paced breathing to lower sympathetic arousal. (3) Family and Team: The presence of family members (if desired) was encouraged to provide emotional anchoring, while the nurse positioned themselves as an active, supportive partner rather than an authoritative evaluator.

#### 2.5.2 Mechanical intervention: visual-feedback respiratory training

Following psychological preparation, patients underwent a 10-min targeted respiratory mechanic warm-up using a KOKA volumetric incentive spirometer (KOKA Medical, China). The goal was to build “muscle memory” for the explosive maneuvers required by the Jaeger spirometer. Inspiratory phase: Patients were instructed to inhale deeply and steadily, attempting to elevate the visual indicator balls (flow target: 1200 mL/s sustained for ≥2 s). Expiratory phase: Patients practiced rapid, forceful exhalation, simulating the FVC maneuver. Technicians provided real-time corrective feedback regarding lip seal integrity, prevention of glottic closure, and maximization of diaphragmatic excursion. Successful completion of three consecutive optimal maneuvers was required before proceeding to the MBCT testing room.

### 2.6 Outcome measures

To ensure objective assessment, outcome assessors (PFT technicians interpreting the spirometry graphs) and statisticians were blinded to group allocation.

- Procedural Efficiency: The primary outcome was the *first-attempt success rate*, defined strictly as generating at least three acceptable and reproducible FVC maneuvers (variance ≤0.15 L) on the first set of attempts without requiring test cessation and re-instruction (8). The *procedural duration* was defined as in-chamber testing duration and was clocked from the moment the first baseline spirometry began until the final methacholine dose or recovery dose was completed.
- MBCT Positivity: The rate of positive challenge results (AHR confirmation) and the provoking concentration causing a 20% fall in FEV1 (PC20) were recorded. This outcome was used descriptively and was not interpreted as direct evidence of improved diagnostic accuracy.
- Psychological Status: Evaluated using the validated Self-Rating Anxiety Scale (SAS) and Self-Rating Depression Scale (SDS) pre-admission and post-procedure (23). The SDS was included because chronic cough patients frequently experience comorbid depressive symptoms due to prolonged sleep disruption and social embarrassment (24). Although the intervention is acute, assessing depression helps capture the baseline holistic emotional burden and explore short-term negative emotional responses to the testing process, rather than to infer treatment of depressive disorder. Both scales have demonstrated high internal consistency (Cronbach’s alpha >0.80).
- Adverse Events: Documentation of procedure-related somatic symptoms (e.g., hoarseness, palpitations, significant dyspnea requiring prolonged rescue medication).

### 2.7 Statistical analysis

Data analysis was performed using SPSS version 26.0 (IBM Corp., Armonk, NY, USA). Continuous variables were tested for normality using the Shapiro–Wilk test. Normally distributed data were presented as mean ± standard deviation (SD) with 95% confidence intervals (CI). Non-normally distributed data were presented as median (interquartile range). Crucially, to adjust for potential selection bias and temporal/seasonal confounding factors (e.g., differing pollen levels between March and July affecting baseline reactivity), a Propensity Score Matching (PSM) analysis was integrated. A logistic regression model was used to calculate propensity scores based on covariates including age, gender, BMI, education level, and baseline FEV1%. Nearest-neighbor matching without replacement was applied at a 1:1 ratio with a caliper width of 0.2 SD. Comparisons between matched groups were conducted using paired t-tests or Wilcoxon signed-rank tests for continuous variables, and McNemar’s test for categorical variables. A Bonferroni correction was applied for multiple comparisons involving spirometric data to minimize Type I error. Effect sizes for psychological scales were calculated using Cohen’s *d* (small: 0.2, medium: 0.5, large: 0.8). A two-tailed *p*-value < 0.05 was deemed statistically significant.

## 3 Results

### 3.1 Baseline characteristics and propensity score matching

Initially, 93 patients were analyzed (Control: 50; Intervention: 43). Prior to matching, minor discrepancies in baseline education levels and BMI were noted, along with the inherent seasonal disparity. Following 1:1 PSM, 41 well-matched pairs (total n = 82) were generated. The matching successfully balanced all baseline covariates, including age, gender, BMI, education level, and baseline lung function (all *p* > 0.05), thereby establishing a robust foundation for comparative analysis (Table 1). Subsequent results are based on the matched cohort.

**Table 1:** Baseline characteristics of participants after propensity score matching (*n* = 82).

Characteristic	Intervention group (*n* = 41)	Control group (*n* = 41)	Statistic (t/χ²)	*p*-value
Age (years), Mean ± SD	45.2 ± 14.8	47.1 ± 15.2	–0.573	0.568
Gender (Male/Female), *n*	12 / 29	15 / 26	0.540	0.462
BMI (kg/m²), Mean ± SD	23.4 ± 3.2	24.1 ± 2.9	–1.037	0.303
Education level, *n* (%)			0.824	0.364
≤ High School	14 (34.1%)	18 (43.9%)		
> High School	27 (65.9%)	23 (56.1%)		
Baseline FEV1/FVC (%), Mean ± SD	95.8 ± 6.2	94.9 ± 7.1	0.612	0.542

Data matched 1:1 using Propensity Score Matching (caliper=0.2) to adjust for seasonal temporal assignment differences. Independent t-test for continuous variables; χ² test for categorical variables.

### 3.2 Physiological stability during procedure

Hemodynamic parameters (heart rate, blood pressure, SpO2) remained clinically stable across both groups throughout the procedure. Both cohorts experienced mild, expected physiological stress responses (e.g., transient elevations in heart rate post-testing, *p* < 0.001), with no severe deviations, ensuring that the intervention itself did not induce hemodynamic instability (Table 2).

**Table 2:** Comparison of vital signs during the testing procedure.

Parameter	Control group (*n* = 41)	Intervention group (*n* = 41)	*p*-value (between groups)
Heart rate (bpm)			
Pre-procedure	75.8 ± 6.1	74.2 ±5.9	0.231
Post-procedure	86.5 ±7.4*	82.1 ±6.2*	0.004
Blood pressure (systolic, mmHg)			
Pre-procedure	121.4 ±8.2	123.6 ±9.1	0.252
Post-procedure	132.8 ±8.9*	130.5 ±9.8*	0.269
SpO2 (%)			
Pre-procedure	96.5 ±1.1	96.4 ±1.2	0.693
Post-procedure	94.8 ±2.1*	97.5 ±1.3	<0.001

Data are presented as mean ±SD. * Indicates *p* < 0.05 compared to intra-group pre-procedure baseline.

### 3.3 In-chamber procedural efficiency and MBCT positivity

**Figure 2:**
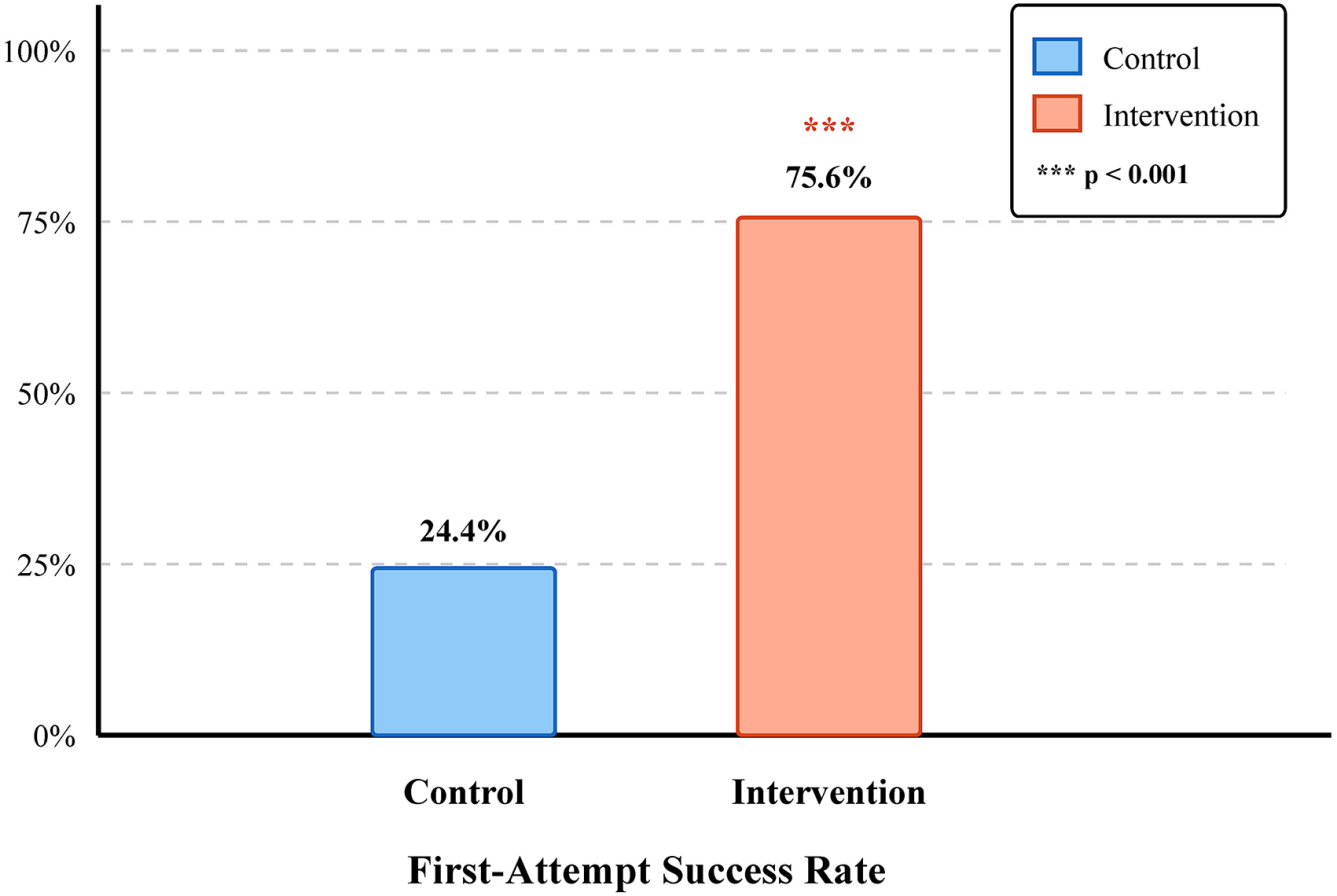
Comparison of first-attempt success rate and MBCT positivity rate. The intervention group showed a higher first-attempt maneuver success rate, whereas the MBCT positivity rate did not differ significantly between groups.

The implementation of communication and respiratory training yielded meaningful improvements in procedural execution. The intervention group exhibited a substantially higher first-attempt success rate compared to the control group (75.6% [95% CI: 60.5, 87.1%] vs. 24.4% [95% CI: 12.4, 40.3%], *p* < 0.001). Concomitantly, the in-chamber testing duration was significantly shorter in the intervention group (11.85 ± 3.12 min vs. 16.90 ± 3.85 min, *p* < 0.001) (Table 3 and Figure 2).

**Table 3:** Procedural efficiency and MBCT diagnostic yield.

**Outcome measure**	**Control group (*n* = 41)**	**Intervention group (*n* = 41)**	**Statistic**	***p*-value**
First-attempt success rate, *n* (%) [95% CI]	10 (24.4%) [12.4, 40.3]	31 (75.6%) [60.5, 87.1]	χ² = 21.57	<0.001
Procedural duration (min), Mean ± SD	16.90 ± 3.85	11.85 ± 3.12	*t* = -6.52	<0.001
MBCT positive result (AHR Confirmed), n (%)	7 (17.1%)	12 (29.3%)	χ² = 1.71	0.190

Procedural duration strictly covers time inside the testing chamber and excludes pre-test psychological and physical intervention time.

Regarding diagnostic outcomes, while the intervention group showed a slightly higher rate of positive MBCT results indicating AHR (29.3% vs. 17.1%), this difference did not reach statistical significance (*p* = 0.190). Because the MBCT positivity rate did not differ significantly between groups, these findings should not be interpreted as evidence of improved diagnostic accuracy.

### 3.4 Optimization of test execution quality

Both groups demonstrated adequate baseline spirometry prior to the challenge. Post-intervention (but pre-methacholine administration), patients in the intervention group generated significantly higher optimal FVC (2.95 ± 0.32 L vs. 2.58 ± 0.35 L, *p* < 0.001) and FEV1 (2.05 ± 0.25 L vs. 1.70 ± 0.28 L, *p* < 0.001) values compared to controls. After applying the Bonferroni correction for multiple testing, these differences remained highly significant. These differences suggest improved maneuver execution and effort mobilization after respiratory training, rather than a physiological improvement in airway function (Table 4).

**Table 4:** Optimization of spirometric maneuver execution (test quality).

**Parameter**	**Control (*n* = 41) [95% CI]**	**Intervention (*n* = 41) [95% CI]**	***p*-value**
Optimal FVC (L)			
Routine baseline	2.25 ± 0.28 [2.16, 2.34]	2.19 ± 0.30 [2.09, 2.29]	0.354
Pre-methacholine (Post-intervention)	2.58 ± 0.35 [2.47, 2.69]	2.95 ± 0.32 [2.85, 3.05]	<0.001
Optimal FEV1 (L)			
Routine baseline	1.31 ± 0.25 [1.23, 1.39]	1.25 ± 0.24 [1.17, 1.33]	0.276
Pre-methacholine (Post-intervention)	1.70 ± 0.28 [1.61, 1.79]	2.05 ± 0.25 [1.97, 2.13]	<0.001

Increases signify enhanced physical effort, glottic control, and procedural execution required prior to methacholine provocation, not physiological disease cure. *P*-values (between-group comparisons at specific time points) remain significant after Bonferroni correction.

### 3.5 Psychological impact

Baseline psychological evaluations revealed moderate anxiety across the cohort, typical for patients undergoing diagnostic testing for chronic persistent cough. Post-procedure, the intervention group exhibited significantly lower anxiety (SAS: 35.10 ± 7.85 vs. 39.50 ± 8.12, *p* = 0.015) and depression scores (SDS: 30.05 ± 5.90 vs. 35.80 ± 6.25, *p* < 0.001) compared to controls. The intervention yielded a medium effect size for anxiety reduction (Cohen’s d = 0.55) and a large effect size for reduction in short-term negative mood or distress (Cohen’s d = 0.94), suggesting a potential psychological buffering effect of the COMFORT model during MBCT (Table 5).

**Table 5:** Psychological assessment (SAS and SDS scores).

**Scale**	**Control group (*n* = 41)**	**Intervention group (*n* = 41)**	***p*-value**	**Cohen's *d***
SAS (Anxiety)				
Pre-admission	58.1 ± 8.0	58.6 ± 8.2	0.778	–
Post-procedure	39.5 ± 8.1	35.1 ± 7.8	0.015	0.55
SDS (Depression)				
Pre-admission	56.5 ± 7.1	57.2 ± 6.8	0.648	–
Post-procedure	35.8 ± 6.2	30.0 ± 5.9	<0.001	0.94

SAS: Self-Rating Anxiety Scale; SDS: Self-Rating Depression Scale. Lower scores indicate better psychological status.

### 3.6 Adverse events

The incidence of procedure-related adverse events was significantly mitigated in the intervention cohort (7.3% vs. 24.4%, *p* = 0.032). Specifically, instances of poor maneuver-induced hoarseness and stress-related palpitations were noticeably reduced. No severe bronchospasm requiring emergency intubation occurred in either group (Table 6).

**Table 6:** Incidence of procedure-related adverse events

**Adverse event type**	**Control group (*n* = 41)**	**Intervention group (*n* = 41)**	***p*-value**
Hoarseness (due to poor vocal cord control)	3	1	0.615*
Stress-induced palpitations	3	0	0.240*
Dyspnea requiring >1 rescue dose	2	1	1.000*
Mild Headache / Dizziness	2	1	1.000*
Total patients with any event, *n* (%)	10 (24.4%)	3 (7.3%)	0.032 (χ²=4.60)

* Fisher's exact test used for individual categories due to low expected counts. Total events were compared using the χ² test.

## 4 Discussion

This study is among the first to integrate a structured psychological framework (COMFORT model) with acute mechanical respiratory training to enhance the procedural execution of methacholine bronchial challenge tests. By applying a quasi-experimental design adjusted via propensity score matching, we rigorously demonstrated that this dual intervention substantially improves first-attempt success rates, shortens in-chamber testing duration, and mitigates patient anxiety in suspected CVA populations.

### 4.1 Clarification on spirometric improvements: performance vs. physiology

A critical distinction must be drawn when interpreting the spirometric data (FVC, FEV1) in this study. The intervention group demonstrated higher measured FEV1 values compared with controls immediately following the respiratory trainer warm-up. As highlighted by recent literature on spirometry quality control, these robust numerical improvements represent the correction of suboptimal baseline maneuvers through enhanced technique and familiarity (25). This is consistent with observations that a significant proportion of spirometry tests, even in clinical settings, fail to meet all stringent acceptability and repeatability criteria on initial attempts, and performance can improve with practice and feedback (26, 27).

Furthermore, it is important to acknowledge that the observed improvements in the intervention group may partially reflect an intrinsic learning effect. The mechanical respiratory training provided repeated practice and real-time corrective feedback prior to the actual test. Consequently, patients were more familiar with the required maneuvers, which inherently contributed to the enhanced procedural quality and higher first-attempt success rates. Patients with suspected CVA often fail to maximally recruit their respiratory musculature due to apprehension or lack of kinesthetic awareness. The KOKA volumetric trainer bridges this gap by translating the abstract command “exhale forcefully” into a measurable, visual objective. Consequently, the “improved” pulmonary function parameters strictly reflect enhanced patient compliance, superior glottic control, and maximized effort—all of which are absolute prerequisites for generating valid, ERS-compliant spirometric curves before the methacholine challenge begins.

### 4.2 In-chamber procedural efficiency and resource considerations

A clinically important finding of our study is the improvement in in-chamber procedural efficiency. The first-attempt success rate increased from 24.4 to 75.6%, accompanied by an average reduction of 5 min in testing chamber occupation time per patient. In a high-volume pulmonary diagnostic laboratory, an artifact-ruined test not only forces the patient to undergo repeated, exhausting forced expirations but also leads to workflow bottlenecks (27). While we significantly conserved the highly specialized, high-cost time within the spirometry chamber by reducing testing duration by approximately 5 min, it is crucial to recognize that the intervention group received an additional 25 min of pre-test preparation (15 min of COMFORT counseling and 10 min of mechanical training) outside the chamber. Therefore, the reported reduction in testing time should be interpreted with caution regarding real-world total workflow efficiency. Although the intervention optimizes the utilization of expensive spirometry equipment, it necessitates increased nursing and allied health resource allocation in the pre-test phase.

Poor maneuver execution can lead to indeterminate tests or underestimation of airway reactivity, highlighting the necessity of optimal technique (28). Previous implementation studies of structured spirometry and patient education programs also support the value of standardizing testing procedures; however, such evidence should not be conflated with a formal cost-effectiveness analysis in the present study (29). Future studies should formally measure total patient time, nursing time, equipment occupancy, repeat-test rates, and laboratory throughput before drawing conclusions regarding cost-effectiveness.

### 4.3 Psychological buffering via the COMFORT model

Patients facing chronic, unexplained cough inherently experience elevated baseline psychological distress (11). The prospect of pharmacologically induced bronchoconstriction during MBCT may further trigger acute situational anxiety. Our data indicate that integrating the COMFORT model was associated with lower post-procedure SAS and SDS scores, yielding a large effect size in the reduction of SDS scores (Cohen’s *d* = 0.94) and a moderate reduction in anxiety.

By replacing paternalistic medical directives with empathetic, team-based communication (“We are doing this together to find the root of your cough”), the intervention may reduce sympathetic arousal and enhance patients’ perceived procedural control. This may help explain the lower incidence of stress-related symptoms, such as palpitations and throat discomfort or hoarseness, observed in the intervention group. Active patient engagement fosters a sense of procedural control, which is critical for obtaining reliable pulmonary function data, as voluntary effort and cooperation directly influence the quality of spirometric maneuvers (30). This comprehensive psychophysical mechanism is illustrated in Graphical Abstract.

### 4.4 Limitations and future directions

While methodologically rigorous, this study possesses several limitations that warrant acknowledgment. Firstly, the quasi-experimental design, despite utilizing rigorous Propensity Score Matching to control for covariates and seasonal variability, inherently cannot replace the gold standard of a prospective, randomized controlled trial (RCT). Unmeasured confounding factors may still exist. Specifically, the historical control design introduces potential temporal biases. Unmeasured factors such as gradual improvements in staff testing proficiency over time, unrecorded changes in patient referral patterns, or subtle seasonal variations in patient baseline characteristics may have influenced the outcomes despite PSM adjustments. Additionally, the study failed to reach the *a priori* target sample size of 106 patients. This shortfall reduces our statistical precision and warrants cautious interpretation of the secondary outcome analyses. Furthermore, no formal economic analysis was conducted to quantify the total resource utilization; therefore, conclusions regarding resource optimization should be interpreted cautiously. Secondly, this was a single-center study. The unique dynamics of our nursing team might limit the immediate generalizability of the COMFORT model to understaffed peripheral clinics. Thirdly, due to the nature of the intervention, double-blinding was impossible; nurses and patients were aware of the training, potentially introducing a Hawthorne effect. However, the outcome assessors analyzing the spirometric flow-volume loops remained strictly blinded. Finally, our study evaluated acute procedural outcomes; future longitudinal studies are needed to assess if a positive, well-communicated testing experience translates to better long-term adherence to asthma inhaler therapies.

## 5 Conclusion

Integrating empathetic nurse–patient communication with targeted mechanical respiratory training fundamentally enhances the execution quality of methacholine bronchial challenge testing. This protocol should be interpreted as improving maneuver preparation and procedural reliability rather than directly changing underlying airway physiology. The higher first-attempt success rate and shorter in-chamber testing duration suggest that this approach may improve MBCT procedural reliability, but its effects on total workflow efficiency, cost-effectiveness, and diagnostic accuracy require prospective evaluation.

## Data Availability

The original contributions presented in the study are included in the article/supplementary material, further inquiries can be directed to the corresponding author/s.

## Glossary

AHR: Airway hyperresponsiveness
BMI: Body mass index
COMFORT: Communication, Orientation and opportunity, Mindfulness, Family, Ongoing, Reiterative, and Team
CVA: Cough-variant asthma
ERS: European Respiratory Society
FEV1: Forced expiratory volume in 1 s
FVC: Forced vital capacity
GINA: Global Initiative for Asthma
LABA: Long-acting β2-agonists
LAMA: Long-acting muscarinic antagonists
MBCT: Methacholine bronchial challenge testing
PC20: Provocative concentration causing a 20% fall in FEV1
PSM: Propensity score matching
RCT: Randomized controlled trial
SABA: Short-acting β2-agonists
SAMA: Short-acting muscarinic antagonists
SAS: Self-Rating Anxiety Scale
SDS: Self-Rating Depression Scale
SOP: Standard operating procedure
TREND: Transparent Reporting of Evaluations with Nonrandomized Designs
